# Safety, Tolerability, and Effect of Nusinersen in Non-ambulatory Adults With Spinal Muscular Atrophy

**DOI:** 10.3389/fneur.2021.650532

**Published:** 2021-04-16

**Authors:** Bakri Elsheikh, Steven Severyn, Songzhu Zhao, David Kline, Matthew Linsenmayer, Kristina Kelly, Marco Tellez, Amy Bartlett, Sarah Heintzman, Jerry Reynolds, Gary Sterling, Tristan Weaver, Kiran Rajneesh, Stephen J. Kolb, W. David Arnold

**Affiliations:** ^1^Department of Neurology, The Ohio State University Wexner Medical Center, Columbus, OH, United States; ^2^Department of Anesthesiology, The Ohio State University Wexner Medical Center, Columbus, OH, United States; ^3^Department of Biomedical Informatics and Center for Biostatistics, The Ohio State University, Columbus, OH, United States; ^4^Assistive Technology Department, The Ohio State University Wexner Medical Center, Columbus, OH, United States

**Keywords:** adults, SMA, non-ambulatory, nusinersen, safety, MUNE

## Abstract

**Objective:** Investigation of the safety, tolerability, and treatment effect of nusinersen treatment in non-ambulatory adults with spinal muscular atrophy (SMA).

**Methods:** Non-ambulatory individuals, aged 18 years or older with genetically confirmed 5q SMA were enrolled. In participants with spinal fusion, fluoroscopy guided cervical C1–C2 lateral approach was used. Outcomes at 2, 6, 10, and 14 months post-treatment were compared with baseline assessment. Forced vital capacity (FVC) was the primary outcome, and RULM, HFMSE, the modified SMA-FRS, and ulnar nerve electrophysiology [compound muscle action potential (CMAP), single motor unit size, and motor unit number] were secondary. Adverse and serious adverse events and clinically significant vital sign or lab abnormalities were recorded.

**Results:** Results from 12 women and 7 men (mean age: 39.7 ± 13.9, range: 21–64 years) were analyzed. No clinically significant changes of vital signs or laboratory parameters were observed. Five participants were hospitalized for pneumonia. Other adverse events included headache, back pain, cervical injection site pain, and upper respiratory and urinary tract infections. High baseline protein/creatinine ratio without significant change on treatment noted in 4 participants. FVC was feasible in all participants. HFMSE and RULM were not feasible in the majority of participants. FVC and functional outcomes were stable without improvement. CMAP and single motor unit potential sizes showed enlargement while motor unit numbers were stable.

**Conclusions:** Nusinersen, including C1/C2 delivery, was safe overall and well-tolerated. Several outcome measures were limited by floor effect. Overall, treatment resulted in stability of motor outcomes, but motor unit and CMAP size were increased.

## Introduction

Spinal muscular atrophy (SMA) is an autosomal recessive disorder caused by reduced levels of survival motor neuron (SMN) protein occurring in ~1 in 11,000 live births (1, 2). In SMA, there is homozygous loss of *SMN1* gene function, but low levels of full length SMN protein are still produced by the *SMN2* gene which are insufficient for normal motor neuron function (1, 3–7). *SMN2* copy number is a determinant of phenotype, and increased copy number usually results in less severity (1, 6, 8–10).

Nusinersen (Spinraza®) is an intrathecal antisense oligonucleotide therapy that targets SMN2 to increase full length SMN protein. Nusinersen was the first FDA-approved treatment and was approved for all SMA types (11). The approval of nusinersen was based on robust data from infants and children (12–14), but data in adults is more limited (15–22). The goals of this study were to investigate the safety, tolerabilty and treatment effect of nusinersen in older, more severely affected, non-ambulatory individuals with SMA. In a parallel study, we have similarly investigated nusinersen in ambulatory adults with SMA [co-submitted, Elsheikh^1^].

## Materials and Methods

### Study Design

This prospective, open label, observational study conducted at the Ohio State University Wexner Medical Center was approved by the institutional review board. Written informed consent was obtained before enrollment. Study visits were conducted between 06/2017 and 01/2020.

### Study Population

Inclusion criteria included: age >18, confirmed 5q SMA, inability to walk, and insurance approval for nusinersen or qualification for free drug. Exclusion criteria included: history of bacterial meningitis or encephalitis, and investigational treatment for SMA in the last 6 months.

### Study Overview

To determine eligibility, screening baseline assessment was completed within 4 weeks of nusinersen initiation. Participants received intrathecal nusinersen treatment on day 1, 15, 29, and 60 followed by maintenance doses every 4 months. Repeated assessments were completed at 2, 6, 10, and 14 months.

### Procedures and Outcome Measures

**Figure 1:**
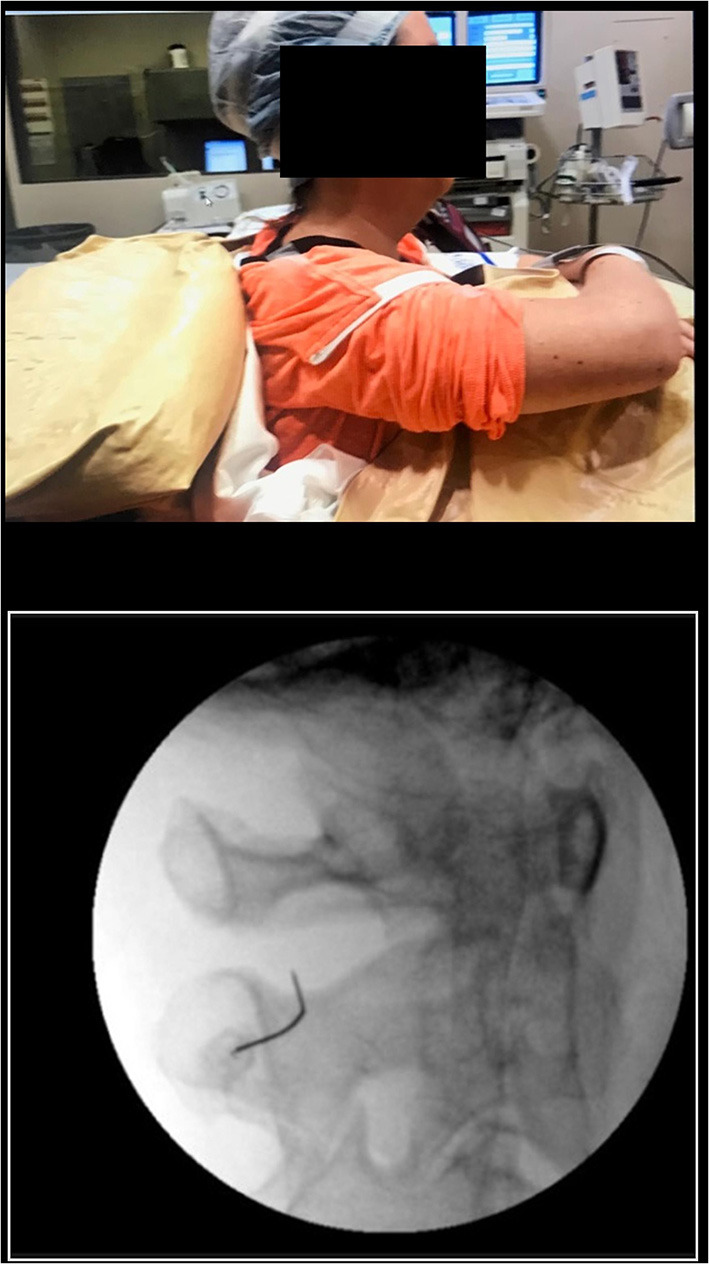
**(A)** The patient seated in preparation for the procedure with an inflatable bag used to maintain posture. **(B)** Fluoroscopy picture showing the position of the anesthesia needle between C1 and C2 spinal lamina.

Nusinersen was delivered by 46 lumbar injections in 7 participants and by 81 cervical C1–2 lateral injections in 12 participants with difficult lumbar access. During 61 injections, individuals were seated in their wheelchair with support of a deflatable bag (Figure 1).

Forced vital capacity (FVC) was the primary outcome. Secondary outcomes included Hammersmith Functional Rating Scale Expanded score (HFMSE), Revised Upper Limb Module (RULM), modified SMA function rating scale (SMA-FRS) score, hand grip, and key pinch strength as well as ulnar compound muscle action potential (CMAP) and single motor unit potential (SMUP) amplitudes and motor unit number estimation (MUNE). Frequency and characteristics of clinically significant vital signs and laboratory abnormalities were assessed. FVC, strength assessments, functional assessments, were performed consistent with methodologies in prior published trials (23, 24). A standardized approach, (http://smaoutcomes.org/hammersmith_manual/cmap.php) was used to measure CMAP amplitude, and multipoint incremental technique was used to obtain average SMUP and calculate MUNE (14).

### Statistical Analysis

Descriptive statistics were performed to summarize the study population including means and standard deviations for the continuous variables and frequencies for the categorical variables. Linear mixed models were used to assess change from baseline of outcome measures across time with random intercepts for each participant. Changes across time were also examined by treatment delivery (lumbar vs. cervical injection). Differences between each time point with baseline and 95% confidence intervals (CI) were reported. Analyses were conducted using the SAS system, version 9.4 (SAS Institute Inc., Cary, NC).

## Results

### Demographics, Disease Severity, Baseline Function, Comorbidities, and Nusinersen Tolerability

A total of 19 participants were enrolled and were assessed up to 14 months following nusinersen initiation. Table 1 describes participant age, gender, SMA genotype and phenotype characteristics, and functional assessments. Mean age of the cohort was 39.7 ± 13.9 years and 12 (63.2%) were women. The majority of participants had 3 copies of *SMN2*. Mean age of onset was 27 ± 34 months, and the cohort was roughly evenly split for SMA 2 and 3. In the participants with SMA type 3 the mean age of loss of ability for ambulation was 25.8 ± 18.3 years. At baseline, SMA-FRS was 11.2 ± 10, Hammersmith 3.5 ± 5.7, and RULM was 12.4 ± 11.5.

**Table 1:** Demographics, genotype and phenotype characteristics, and baseline functional assessment.

**Variable**	**Level**	**Total (*n* = 19)**
Age	Mean (Std) (min, max)	39.7 (13.9) (21.3, 64.8)
Sex	Women	12 (63.2%)
	Men	7 (36.8%)
SMN2 copies	2	1 (5.3%)
	3	16 (84.2%)
	4	2 (10.5%)
Age of onset (months)	Mean (Std) (min, max)	27 (34) (2–156)
SMA type	2	9 (47%)
	3	10 (53%)
Age at loss of ambulation (SMA 3) (years)	Mean (Std) (min, max)	25.8 (18.3) (8, 57)
Baseline SMA-FRS	Mean (Std) (min, max)	11.2 (10) (0, 30)
Baseline hammersmith	Mean (Std) (min, max)	3.5 (5.7) (0, 18)
Baseline RULM	Mean (Std) (min, max)	12.4 (11.5) (0, 37)

Baseline medical comorbidities included: spinal fusion in 10, kidney stones in 5, deep venous thrombosis on anticoagulation in 4, hypertension in 3, diabetes in 1, recurrent pneumonia in 3, and recurrent urinary tract infection (UTI) in 2. During the study, there were 5 pneumonia-related hospitalizations and two musculoskeletal injuries during transfer (rotator cuff injury and femur fracture). Of the 19 participants, 9 were treated with intermittent, non-invasive pressure support, 3 were treated with invasive support, and 7 were not treated with any ventilatory support. For the participants on anticoagulation, agents were held 3–5 days (based on the agent), and for high risk participants a bridge with low molecular weight heparin, coordinated through the primary care physician.

Side effects included transient headache, nausea, dizziness, back and neck pain, UTI, and URI (Lumbar: Table 2 and Cervical: Table 3). No clinically significant vital sign abnormalities were noted. High baseline protein/creatinine ratio without significant change on treatment was noted in 4 participants. One participant discontinued related to enrollment in another interventional study.

**Table 2:** Adverse events lumbar injections.

	**Loading 1**	**Loading 2**	**Loading 3**	**Loading 4**	**6 months**	**10 months**	**14 months**	**Percent of all injections**
**LUMBAR (7 subjects# received total of 46 injections)**								
Headache	3 (A, B, C)	2 (A, B)	1 (A)	2 (A, C)	2 (A, B)	2 (B, D)		26%
Back pain	3 (A, C, E)	2 (A, C)	2 (A, C)	2 (A, C)	1(C)	2 (A, C)	1 (A)	28.2%
Nausea	1 (E)							2.1%
Vomiting								
Dizziness								
Neck Pain	1 (F)		1 (F)					4.3%

*#Letters indicate individual participants*.

**Table 3:** Adverse events lumbar injections.

	**Loading 1**	**Loading 2**	**Loading 3**	**Loading 4**	**6 months**	**10 months**	**14 months**	**Percent of all injections**
**CERVICAL (12 subjects# received total of 81 injections)**								
Headache	4 (G, H, I, J)	3 (H, I, K)	3 (I, L, M)	4 (I, M, N, O)	3 (I, P, Q)	5 (H, I, M, N, O)	3 (H, I, N)	30.8%
Back pain	2 (J, M)	2 (J, M)	3 (G, J, M)	1 (M)		2 (M, R)	1 (S)	13.5%
Nausea			1 (I)					1.2%
Vomiting								
Dizziness					1 (I)			1.2%
Neck Pain	3 (P, R)	2 (P, R)	2 (P, R)	2 (P, R)	1 (R)	2 (I, Q)	1 (R	16.0%)

*#Letters indicate individual participants*.

### Prospective Outcome Assessments

Table 4 shows longitudinal outcome assessments in participants at baseline and estimated change to baseline at 2, 6, 10, and 14 months. Table 4 indicates the number of participants assessed at each time point, and for most outcomes, 12 participants were studied at all time points. Two indices of respiratory muscle function were assessed, FVC and NIF. FVC, the primary outcome, and NIF showed no significant change at any time point compared with baseline. FVC and NIF results suggested a pattern of stable ventilatory muscle function over the 14 month period of the study.

**Table 4:** Longitudinal change of outcomes.

**Measure**	**Time/Comparison**	***N***	**Estimate (baseline/change from baseline)#**	**95% CI**	***p*-value**
**Pulmonary function**					
Actual FVC	Baseline	19	1.94	1.3, 2.58	
	2 months–baseline	17	−0.02	−0.11, 0.08	0.7296
	6 months–baseline	19	−0.02	−0.11, 0.07	0.6628
	10 months–baseline	17	−0.02	−0.11, 0.07	0.6762
	14 months–baseline	12	0.02	−0.09, 0.12	0.7733
NIF	Baseline	19	−37.84	−46.79, −28.89	
	2 months–baseline	17	−7.46	−17.88, 2.96	0.1571
	6 months–baseline	18	−5.74	−15.97, 4.49	0.2658
	10 months–baseline	17	−5.06	−15.47, 5.34	0.3343
	14 months–baseline	12	−9.14	−20.83, 2.55	0.1232
**Functional scales**					
Hammersmith	Baseline	19	3.47	0.01, 6.93	
	2 months–baseline	18	0.77	−0.29, 1.83	0.1515
	6 months–baseline	19	0.74	−0.3, 1.78	0.1624
	10 months–baseline	19	0.32	−0.73, 1.36	0.5468
	14 months–baseline	12	0.11	−1.11, 1.32	0.8606
SMA-FRS	Baseline	19	11.16	6.61, 15.7	
	2 months–baseline	18	−0.26	−1.28, 0.77	0.6510
	6 months–baseline	19	−0.58	−1.59, 0.43	0.2551
	10 months–baseline	19	−0.58	−1.59, 0.43	0.2551
	14 months–baseline	14	−0.98	−2.1, 0.13	0.1306
RULM	Baseline	19	12.42	6.91, 17.93	
	2 months–baseline	18	1.31	0.24, 2.39	0.0171
	6 months–baseline	19	0.89	−0.16, 1.95	0.0946
	10 months–baseline	19	0.95	−0.1, 2	0.0771
	14 months–baseline	12	0.27	−0.96, 1.5	0.6637
**Strength measurement**					
Key pinch	Baseline	17	0.55	0.05, 1.05	
	2 months–baseline	18	0.18	0.07, 0.29	0.0019
	6 months–baseline	19	0.1	−0.01, 0.21	0.0748
	10 months–baseline	19	0.1	−0.005, 0.21	0.0617
	14 months–baseline	12	0.01	−0.11, 0.14	0.8484
Handgrip	Baseline	17	1.31	0.39,2.23	
	2 months–baseline	18	0.2	−0.21,0.61	0.3405
	6 months–baseline	19	0.43	0.03, 0.84	0.0377
	10 months–baseline	19	0.13	−0.28, 0.54	0.5236
	14 months–baseline	12	0.11	−0.36, 0.57	0.6514
**Electrophysiological measures of motor unit connectivity**					
CMAP	Baseline	19	2.54	1.34, 3.74	
	2 months–baseline	18	0.12	−0.14, 0.38	0.3446
	6 months–baseline	19	0.22	−0.04, 0.47	0.0949
	10 months–baseline	19	0.29	0.04, 0.55	0.0238
	14 months–baseline	13	0.32	0.03, 0.61	0.0308
**For the measurements below (SMUP and MUNE), only 13 patients were included in the analysis**					
SMUP	Baseline	12	98.49	72.52, 124.46	
	2 months–baseline	11	2.69	−1.35, 6.72	0.1852
	6 months–baseline	12	3.31	−0.6, 7.23	0.0949
	10 months–baseline	12	5.09	1.18, 9.01	0.0122
	14 months–baseline	6	6.98	1.99, 11.98	0.0074
MUNE	Baseline	12	40	19.44, 60.56	
	2 months–baseline	11	−1.25	−4.13, 1.62	0.3816
	6 months–baseline	12	−1.17	−3.95, 1.62	0.4020
	10 months–baseline	12	0.08	−2.7, 2.87	0.9520
	14 months–baseline	6	−1.25	−4.81, 2.31	0.4807

*Statistical method: linear mixed models were used to explore the change of these measures across time with random intercepts for each participant. Difference between each time point with baseline and 95 % CI were reported. #Positive values indicate increase compared to baseline, and negative values indicate decrease from baseline at each time point (2, 6, 10, and 14 months)*.

Two scales of disease severity were investigated in this study, HFMSE and SMA-FRS. HFMSE was not scorable (scored as 0) in 13 of the 19 participants due to phenotypic severity. In the remaining 6 participants, HFMSE showed no significant change over the 14 months of the study. SMA-FRS was scorable in all 19 but showed no significant change with treatment.

Upper limb function was assessed with the RULM. RULM was not scorable in 6 participants due to disease severity, but longitudinal assessment showed a transient significant improvement at 2 months (11% increase from baseline). Similarly, measures of upper limb muscle strength were assessed including key pinch and grip strength. Key pinch showed a transient increase at 2 months (33% increase from baseline). Similarly, hand grip showed a transient increase at 6 months (33% increase from baseline).

Electrophysiological measures of motor unit function including CMAP, average SMUP, and MUNE were recorded to understand the impact of nusinersen on motor unit number and connectivity. CMAP amplitude (shown in mV in Table 4) was obtained in all 19 participants and showed significant increases at 10 (11% increase from baseline) and 14 months (13% increase from baseline). Average SMUP amplitude (shown in μV in Table 4) was obtained in 13 participants. Average SMUP amplitude, which can be used as an index of collateral sprouting and reinnervation, was significantly increased at 10 months (5% from baseline) and 14 months (7% from baseline). In the 13 participants who underwent average SMUP assessment, MUNE was calculated (MUNE = CMAP amplitude/Average SMUP amplitude). In contrast to CMAP and SMUP, MUNE showed no significant change.

### Comparison of Lumbar vs. Cervical Intrathecal Approach

To explore the possibility of an impact of injection route on the effect of nusinersen, we also explored changes in outcome assessments stratified by injection route (Table 5). As expected, participants that required cervical injections were more severely affected. For FVC, the primary outcome, neither group showed significant changes at any time point (compared to baseline), but the change of FVC over time differed by injection route (*p*-value for interaction = 0.014). HFMSE, SMA-FRS, and RULM showed no significant differences over time with respect to treatment delivery (all *p*-values for interaction > 0.05). Key pinch did not change over time for participants undergoing cervical injections, but participants that underwent lumbar injection showed improvements at earlier time points at 2 months (~42% increase from baseline) and 6 months (28% increase from baseline) (*p*-value for interaction = 0.0003).

**Table 5:** Longitudinal change of outcomes stratified by cervical or lumbar.

**Measure**	**Time**	**Cervical**			**Lumbar**		
		**Estimate**	**95% CI**	***p*-value**	**Estimate**	**95% CI**	***p*-value**
**Primary outcome**							
Actual FVC	Baseline	1.34	0.91, 1.76		2.97	1.47,4.46	
	2 month–baseline	0.03	−0.04, 0.09	0.4206	−0.09	−0.32, 0.14	0.4223
	6 month–baseline	0.03	−0.03, 0.09	0.3333	−0.1	−0.32, 0.11	0.3252
	10 month–baseline	0.002	−0.06, 0.07	0.9586	−0.05	−0.27, 0.16	0.6146
	14 month–baseline	−0.03	−0.1, 0.03	0.3131	0.2	−0.09, 0.49	0.1688
*p*-value for interaction	0.0140						
**Functional scales**							
HFMSE	Baseline	0.83	−1.06, 2.73		8	−0.48, 16.48	
	2 month–baseline	0.03	−0.08, 0.13	0.6073	2	−0.97, 4.97	0.1757
	6 mo–baseline	0	−0.1, 0.1	1.0000	2	−0.97, 4.97	0.1757
	10 month–baseline	0.08	−0.02, 0.19	0.1110	0.71	−2.25, 3.68	0.6219
	14 month–baseline	0.03	−0.09, 0.15	0.6444	0.12	−3.48, 3.71	0.9460
*p*-value for interaction	0.2009						
SMA-FRS	Baseline	6.92	4.12, 9.71		18.43	7.96, 28.89	
	2 month–baseline	−0.2	−1.59, 1.17	0.7588	−0.43	−3.84, 2.98	0.7967
	6 month–baseline	0	−1.34, 1.34	1.0000	−1.57	−4.98, 1.84	0.3494
	10 month–baseline	−0.5	−1.84, 0.84	0.4557	−0.71	−4.12, 2.69	0.6681
	14 month–baseline	−1.26	−2.69, 0.16	0.0805	−3.48	−7.29, 0.33	0.0714
*p*-value for interaction	0.5664						
RULM	Baseline	8	2.61, 13.39		20	8.24, 31.76	
	2 month–baseline	1.3	0.27,2.34	0.0151	1.29	−1.17, 3.74	0.2890
	6 month–baseline	1.25	0.24, 2.26	0.0164	0.29	−2.17, 2.74	0.8113
	10 month–baseline	1.42	0.41, 2.42	0.0071	0.14	−2.31, 2.6	0.9049
	14 month–baseline	0.86	−0.3, 2	0.1426	−0.86	−3.84, 2.11	0.5526
*p*-value for interaction	0.5394						
**Physical function**							
key_pinch	Baseline	0.24	−0.07, 0.56		1.05	−0.22, 2.33	
	2 month–baseline	0.025	−0.03, 0.08	0.3677	0.45	0.19, 0.71	0.0020
	6 month–baseline	−0.005	−0.06, 0.05	0.8591	0.29	0.03, 0.56	0.0307
	10 month–baseline	0.05	−0.001, 0.11	0.0551	0.21	−0.05, 0.47	0.1140
	14 month–baseline	0.004	−0.06, 0.07	0.8814	0.01	−0.29, 0.32	0.9216
*p*-value for interaction	0.0003						
**Electrophysiological measures of motor unit connectivity**							
CMAP	Baseline	1.75	0.42, 3.09		3.89	1.65, 6.12	
	2 month–baseline	0.11	−0.005, 0.22	0.0603	0.17	−0.5, 0.84	0.5995
	6 month–baseline	0.12	0.009, 0.22	0.0349	0.39	−0.28, 1.05	0.2436
	10 month–baseline	0.05	−0.06, 0.16	0.3550	0.71	0.05, 1.38	0.0374
	14 month–baseline	0.08	−0.04, 0.2	0.1867	0.82	0.01, 1.63	0.0468
*p*-value for interaction	0.0197						
**For the measurements SMUP, only 13 patients were included in the analysis**							
SMUP	Baseline	90	58.54, 121.46		106.98	55.29, 158.67	
	2 month–baseline	0.73	−2.98, 4.44	0.6806	4.77	−1.5, 11.04	0.1270
	6 month–baseline	0.17	−3.32, 3.65	0.9205	6.46	0.18, 12.73	0.0444
	10 month–baseline	−0.17	−3.65, 3.32	0.9205	10.36	4.08, 16.63	0.0028
	14 month–baseline	0.43	−4.02, 4.88	0.8403	13.56	5.57, 21.56	0.0023
*p*-value for interaction	0.0166						

For electrophysiological outcomes, CMAP showed a significant difference between injection routes over time (*p*-value for interaction = 0.0197). In participants who received cervical injections there were significant changes at 6 months (7% increase from baseline) compared with baseline, and in participants undergoing lumbar injection there were significant increases at 10 (18% increase from baseline) and 14 months (~21% increase from baseline). SMUP also showed a significant difference between injection routes over time (*p*-value for interaction = 0.0166). Participants undergoing lumbar injections showed significant increases at 6 months (6% increase from baseline), 10 months (10% increase from baseline), and 14 months (13% increase from baseline), but participants undergoing cervical injections showed no significant change at any time point.

## Discussion

This study demonstrated several important and relevant findings for the management of severely affected, non-ambulatory adults with SMA, a group that has been largely excluded from prior trials. Our study showed that nusinersen was well-tolerated. Overall, 10–14 months of nusinersen treatment resulted in stability of outcome measures of ventilatory muscle function (FVC and NIF), muscle strength and function. Our primary outcome, FVC, did not demonstrate improvement, but lack of decline may suggest a mild positive effect based on the expected decline noted in a recent large natural history study that showed −1.32 to −0.67% predicted FVC reduction/year in patients with type 2a−3a SMA (25). Our results provide evidence that commonly used SMA outcome measures, such HFMSE, are not optimized for severely affected individuals.

One of the most remarkable findings of our study were increases of CMAP and SMUP amplitudes. Prior studies have shown effects of age and function on treatment responses (1). Preclinical studies showed preservation of motor neuron and ventral root counts and motor unit numbers with early treatment while delay resulted in improved output from the remaining motor neurons (i.e., increased collateral sprouting) (26, 27). Increases in CMAP and SMUP without changes in MUNE are consistent with these preclinical findings and were also noted in our parallel ambulatory adult study^1^. One prior study in children with later onset SMA treated with nusinersen, showed decreased MUNE and stable CMAP, but data in this study showed variability possibly reflecting the difficulty of electrophysiological assessments in children (14). Another recent study used the technique of MScanFit MUNE to investigate motor unit response in children undergoing nusinersen treatment and showed recovery of smaller motor units (28). This is in contrast to what we show here in that adults undergoing nusinersen demonstrated enlargement of average motor unit size (amplitude) without change in MUNE. Whether these differences were related to differences in technique or due to differences in biological response related to age (children vs. adults) deserves further attention in future studies. Perhaps a future study could investigate electrophysiological responses to SMN restoration using both techniques (MScanFit MUNE and multipoint incremental MUNE) in children and adults.

It is worth pointing out that our cohort involved participants with more severe phenotypes as compared with other recent investigations (17, 19). In severely weak patients, scoliosis and spinal fusion require alternative routes for intrathecal access. We show that our alternate C1/C2 lateral approach was well-tolerated. We explored whether route of intrathecal delivery might impact outcomes differently. Interestingly, delivery via cervical injection resulted in more consistent improvements of RULM. It seems plausible that cervical delivery could result in greater SMN induction in upper cervical spinal cord regions and thus greater impact on proximal upper limbs. Interestingly, comparisons of functional readouts from the lower cervical regions (C8/T1 myotomes: key pinch, CMAP, and SMUP) were more impacted following lumbar injection. So these findings could also simply reflect differences in the sensitivity of these outcomes. Another important consideration in regards to changes in the RULM is the possibility of a learning effect which could result in changes irrespective of nusinersen effect, and this effect cannot be excluded due to the lack of a control group (12). From the current studies, it is not clear what might be explaining the discrepancies between treatment routes and outcome responses, but the impact of route of delivery deserves more attention in future studies.

Our study supports the tolerability and suggests a positive impact of nusinersen in weaker adults with SMA. Yet, generalizability of the findings should be considered in the context of our study's small sample size, open label design, and limited longitudinal data. Future studies should focus on improvement of outcome measures for this population and understanding of the impact of different routes of intrathecal delivery.

## Data Availability Statement

The original contributions presented in the study are included in the article/supplementary material, further inquiries can be directed to the corresponding author/s.

## Ethics Statement

The studies involving human participants were reviewed and approved by the Ohio State University. The patients/participants provided their written informed consent to participate in this study.

## Author Contributions

BE: drafting/revision of the manuscript for content, including medical writing for content, major role in the acquisition of data, study concept or design, and analysis or interpretation of data. SS: drafting/revision of the manuscript for content, including medical writing for content, and study concept or design, and analysis or interpretation of data. SZ, DK, SK, and WA: drafting/revision of the manuscript for content, including medical writing for content, study concept or design, and analysis or interpretation of data. ML, MT, AB, SH, JR, and GS: drafting/revision of the manuscript for content, including medical writing for content, and major role in the acquisition of data. KK: drafting/revision of the manuscript for content, including medical writing for content, major role in the acquisition of data, and analysis or interpretation of data. TW and KR: drafting/revision of the manuscript for content, including medical writing for content, and analysis or interpretation of data. All authors contributed to the article and approved the submitted version.

## Conflict of Interest

BE received compensation for consulting from Biogen, Genentech, Argenx, and Stealth Bio-therapeutics. TW received compensation for consulting from Medtronic, Inc and PainTeq. SK received compensation for consulting from Genentech, AveXis, and Biogen. WA received compensation for consulting for La Hoffmann Roche and Genentech. The remaining authors declare that the research was conducted in the absence of any commercial or financial relationships that could be construed as a potential conflict of interest.

## Footnotes

^1^Elsheikh B. Safety, *Tolerability, and Effect of Nusinersen in Ambulatory Adults With Spinal Muscular Atrophy*. *Front. Neurol*. (2021).
